# ‘Why Did They Migrate Here’?: A Qualitative Descriptive Study Exploring Nurses' Motivations for Migration and Regional Relocation

**DOI:** 10.1111/jan.16620

**Published:** 2025-01-16

**Authors:** Princess Villamin, Violeta Lopez, Deependra Kaji Thapa, Michelle Cleary

**Affiliations:** ^1^ School of Nursing, Midwifery and Social Sciences CQUniversity Sydney Australia; ^2^ Department of Epidemiology and Biostatistics School of Public Health, Indiana University Bloomington Indiana USA

## Abstract

**Aim:**

To explore migrant nurses' intrinsic and extrinsic motivations for migration and regional relocation.

**Design:**

A qualitative descriptive study.

**Methods:**

Semi‐structured interviews were conducted among 17 migrant nurses working in a hospital in regional Australia. Inclusion criteria were current employment as a nurse at the study site, obtaining an initial nursing qualification in a different country, and migrating to regional Australia within the last 7 years. Data were analysed using a thematic approach, informed by the self‐determination theory.

**Results:**

One overarching theme, seeking meaningful endeavours, was identified, with subthemes: pride in nursing, duty to family and personal satisfaction. Extrinsic motivations included financial responsibilities, visa security, professional nursing recognition and fulfilment, filial piety, family unification and overall safety and lifestyle, whereas intrinsic motivations included being a nurse and travelling.

**Conclusion:**

Nurses' motivations for migration are complex and driven by intrinsic and extrinsic factors. Extrinsic motivations may lead to self‐endorsed behaviour (autonomous motivation) if they align with personal goals or values, such as family importance or regard for one's profession. All intrinsic motivations also lead to autonomous motivation, linked to well‐being and workplace retention.

**Implications for the Profession:**

This study's findings may inform organisations in source and host countries to design work conditions that foster retention. Source countries can use these insights to address the professional limitations experienced by their nurses. Host countries may design targeted strategies that promote autonomous motivation among migrant nurses, thereby enhancing job satisfaction, well‐being and retention.

**Impact:**

This study provides insight into experiences contributing to migrant nurses' relocation decisions. Practising within the scope of their professional training, performing their duties in a fulfilling way, and meeting their family obligations through reasonable pay or benefits may support nurses in remaining autonomously motivated.

**Reporting Method:**

COREQ reporting was adhered to.

**Patient or Public Contribution:**

No patient or public contribution.

## 1 Introduction

Motivation can be described as the intention underlying action and can be classified as intrinsic and extrinsic (Ryan, Bradshaw, and Deci 2019). Intrinsic motivation stems from an inherent interest and satisfaction from the activity, whereas extrinsic motivation stems from the rewards and consequences of engaging in the activity (Ryan and Deci 2020). In the literature, nurses' motivations have been explored and distinguished between extrinsic and intrinsic in the context of overall work motivation (Toode et al. 2014), well‐being (Kohnen et al. 2023), and retention (Satoh, Watanabe, and Asakura 2018), not nurse migration.

Given the current nursing shortages and increasing migration of nurses to, from, and between host and source countries, exploring intrinsic and extrinsic motivations provides insight into areas that may be meaningfully impacted by policy change, given that motivation has been identified as an attribute of nurse migration and retention (Efendi et al. 2019; Freeman et al. 2012). Identifying and distinguishing between intrinsic and extrinsic motivations are crucial as they arise from different psychological processes, resulting in distinct long‐term outcomes, including retention or stepwise (onward) migration. This paper is part of a larger study to understand motivations and identify factors affecting the motivations of migrant nurses that lead to regional workplace retention. Migrant nurses' intrinsic and extrinsic motivations to migrate are reported in this paper, whereas factors relating to the decision to stay are explored in a companion paper (under review).

## 2 Background

Intrinsic motivation involves engaging in activities ‘for their own sake’ or from the pleasure of engaging in the activity rather than from the expectation of rewards (Chirkov et al. 2007; Ryan and Deci 2020, 101860). Extrinsic motivation is engagement in activities for reasons other than deriving enjoyment. Motivation is described along a continuum, with amotivation on one end, followed by extrinsic motivation and intrinsic motivation on the other end (Gagné and Deci 2005). Amotivation is the lack of motivation or intention to engage in an activity or behaviour due to a lack of interest or felt competence to perform (Ryan and Deci 2020; Van den Broeck et al. 2021). There are four types of extrinsic motivation depending on the degree to which individuals attempt to regulate or transform their behaviour into personally endorsed actions: (1) external, wherein behaviour is contingent on reward or punishment; (2) introjected, wherein behaviour is dependent on self‐esteem or avoidance of feelings of loss, shame, or guilt for engaging (or not engaging) in the activity; (3) identified, wherein behaviour is self‐endorsed as the individual recognises the value of the activity and (4) integrated, wherein behaviour is self‐endorsed as the activity is in congruence with one's goals, interests, and values (Deci, Olafsen, and Ryan 2017; Ryan and Deci 2020; Van den Broeck et al. 2016). Behaviour is often described as controlled when the motivation stems from internal or external ‘controls’ or pressures, such as being controlled by financial rewards (external) or self‐esteem (introjected). On the other hand, behaviour is referred to as autonomous when it is self‐endorsed as the activity is seen as necessary (identified), aligns with one's goals, values or interests (integrated), or is inherently enjoyable (intrinsic) (Gagné and Deci 2005). Higher or more autonomous forms of motivation, such as identified, integrated, or intrinsic motivation, may result in favourable outcomes, including job satisfaction, commitment, engagement, decreased turnover intention, burnout, and absenteeism (Gagné and Deci 2005; Gagné et al. 2015; Rigby and Ryan 2018; Van den Broeck et al. 2021).

For example, nurses may be autonomously motivated to care for the sick because they enjoy the act of nursing (intrinsic), as being a nurse is central to their identity or aligned with their values (integrated), or to meet their goal of enhancing patient comfort and well‐being (identified), despite some nursing tasks not being inherently enjoyable. Then again, nurses' motivations may be thought of as controlled when they feel internal pressures to achieve self‐worth from ‘being a good nurse’ (introjected) or to obtain rewards such as pay (external) (Gagné and Deci 2005). The demarcation of motivations on the continuum does not mean that nurses are only either intrinsically or extrinsically motivated. Rather, motivations can simultaneously exist, especially when work is involved, as work is inherently focused on compensation and rewards (Moran et al. 2012; Howard et al. 2016). The value of breaking down motivation levels may elucidate why some nurses do what they do—in the context of migration—why some migrate whilst others do not, and why some nurses who migrated choose to stay in their initial host country whilst others proceed to migrate onwards.

The literature identifies a typical pattern of migration from low‐income (source) to middle‐income and high‐income (host) countries, with motivations for migration varying from financial to organisational and personal reasons (Konlan, Lee, and Damiran 2023; Villamin et al. 2024a). However, the current migration pattern has become more dynamic and less predictable, highlighting retention issues among source and host countries, especially in regional, remote, and rural areas (Jones, Rahman, and Jiaqing 2019; Pressley et al. 2022; Tejero et al. 2022). The findings of this study inform our understanding of the motivation to migrate to a host country, followed by relocation to a regional area. Although there is supporting evidence explaining motivations for migration from low‐income to middle‐income and high‐income countries, research describing motivations for nurses migrating between middle‐income and high‐income countries or between high‐income countries is scarce. Additionally, more needs to be understood about the events preceding the decision to migrate.

Published research on nurse motivations for migration is primarily focused on extrinsic motivations such as financial and professional opportunities (Konlan, Lee, and Damiran 2023). This view may undermine the interaction of other sources of motivation that contribute to a decision to migrate. Migration is a significant life transition, often involving a complicated process wherein individuals may experience a myriad of challenges, such as cultural shock, financial insecurity, professional challenges, and discrimination both in the community and the workplace, often affecting their physical and psychological well‐being (Tan and Cebulla 2022; Torres Fernández et al. 2017). This may imply that since migration often requires one to leave behind established social networks, friends, and family and subject oneself to unfamiliar cultural, social, and professional norms, it may not be an activity done inherently without any form of extrinsic motivation. Although a sense of adventure and travel may intrinsically drive migration (Despotovic, Hutchings, and McPhail 2022), migration may also be considered a by‐product of contextual factors and experiences that do not satisfy nurses' intrinsic and extrinsic motivations, such as inadequate financial compensation, limited professional autonomy, or insufficient professional recognition (Pressley et al. 2022; Toyin‐Thomas et al. 2023). Positive migration outcomes rely on higher forms of motivation (Yang, Zhang, and Sheldon 2018), which supports the need to explore nurses' intrinsic and extrinsic motivations and how these contribute to migration.

## 3 The Study

### 3.1 Aim

This study aims to understand the motivations and factors affecting the motivations of migrant nurses that lead to regional workplace retention. The research question is: How do migrant nurses describe their intrinsic and extrinsic motivations for migration? This paper reports on migrant nurses' intrinsic and extrinsic motivations that contributed to their initial migration to the host country, including relocation to the regional area. Other factors describing migrant nurses' needs and experiences after relocation and how these lead to regional workplace retention are reported elsewhere (paper under review).

### 3.2 Design

This study used a qualitative descriptive approach, which allowed researchers to stay close to participants' experiences and gain a comprehensive understanding of these (Doyle et al. 2020; Sandelowski 2010). This approach enables researchers to obtain first‐hand descriptions of participants' perceptions, which is appropriate to meet the study's aim.

### 3.3 Theoretical Framework

The self‐determination theory (SDT) was adopted as a theoretical framework for this study. SDT is a broad motivation framework highlighting that individuals have inherent basic psychological needs for autonomy, competence, and relatedness, which are fundamental to well‐being, development, and optimal functioning, leading to higher forms of motivation (Legault 2017; Ryan and Deci 2000). SDT proposes that the quality of motivation, influenced by the fulfilment of autonomy, competence, and relatedness, drives the level of persistence with desired behaviour (Manninen et al. 2022; Ryan and Deci 2000). SDT is supported by robust evidence in its applicability across cultures and has provided a valuable framework for organisational research and understanding motivation in the workplace in relation to various workplace outcomes such as career satisfaction, work engagement, and turnover intention (Hwang, Song, and Ko 2022; Ni et al. 2023; Onyishi et al. 2019; Van den Broeck et al. 2021).

### 3.4 Study Setting and Recruitment

The study was conducted in one hospital in regional Australia. The state where this region is located has one of the highest proportions of migrants in Australia, with one of the highest grant rates of temporary working and permanent visas to migrant nurses (Australian Bureau of Statistics 2021; Department of Home Affairs 2022). However, the state has one of the lowest full‐time equivalent nurses per 1000 population in Australia and an uneven distribution of nurses in regional and metropolitan areas (Australian Institute of Health and Welfare 2023).

Participants were recruited via purposive and snowball sampling. These sampling strategies were essential for the study as purposive sampling enabled researchers to purposefully select participants who may provide information‐rich and abundant data relevant to the topic (Staller 2021), whereas snowball sampling allowed researchers to negate initial low recruitment response rates and locate participants who were not easily accessible (Leighton et al. 2021). Participants were nurses who (a) were currently employed at the site of study, (b) obtained their initial nursing qualification in a different country and (c) migrated to regional Australia within the last 7 years from another country or within Australia.

### 3.5 Data Collection

Data were collected via individual, semi‐structured interviews conducted by a single interviewer (P.V.) for consistency. This enabled the researcher to obtain narrative responses and explore issues in‐depth through probing, supporting the depth of data (Bradshaw, Atkinson, and Doody 2017). The research team developed an interview guide based on the research literature and SDT (Ryan and Deci 2000). The interview questions were structured to address concepts from the SDT; for example, the question ‘What factors motivate you at work?’ may reflect extrinsic motivation if participants are motivated by salary, introjected regulation if participants are motivated by ego or self‐esteem, or intrinsic motivation if participants are motivated by an inherent desire to look after others.

This paper reports on interview responses to the following eight questions from the semi‐structured interview guide: Why did you migrate? What events or experiences made you decide to move? What/Who motivated you to migrate? What factors motivate you at work? How does being a nurse make you feel? How important is being a nurse to you? If you had another option, would you continue to work as a nurse—why or why not? When appropriate, further questions were asked to probe the participants' responses. Demographic questions were also asked at the beginning of the interview. Responses to other questions in the interview guide are reported elsewhere (paper under review).

Before data collection, pilot interviews were conducted (P.V.) and reviewed (M.C.) to ensure the participants adequately understood the questions. The pilot interviews were not included in the final interview count. Participants were given a participant information sheet and consent form to be returned to the interviewer (P.V.) before scheduling the interview. Depending on participant preference, interviews were conducted via telephone or MS Teams between November 2023 and February 2024. Most participants opted to participate outside of work hours despite blanket organisational approval for participation during work hours. Interviews were between 45 and 60 min, audio‐recorded, transcribed verbatim, and proofread for accuracy. Data collection and analysis were concurrent. Data collection ceased when no further codes were developed from repeated rounds of coding, and the researchers were satisfied with the adequacy of their data, thereby achieving data saturation. Data saturation is crucial in qualitative research and is supported by data adequacy, appropriateness, and redundancy (Braun and Clarke 2021; Cleary, Horsfall, and Hayter 2014; Morse 1995).

### 3.6 Data Analysis

Data were analysed using reflexive thematic analysis (Braun and Clarke 2006, 2022) and an inductive approach, which allowed the development of codes from the participants' responses. Braun and Clarke (2006, 2022) endorse six steps to conducting reflexive thematic analysis, which include (1) data familiarisation, (2) initial coding, (3) generating initial themes, (4) developing and reviewing themes, (5) refining, defining, and naming themes and (6) writing the report.

To begin, transcripts were read and re‐read against the audio recordings to ensure accuracy and to ‘hear something said’ (Sandelowski 2000, 373). Note‐taking during the familiarisation phase involved annotating initial thoughts and ideas. Following familiarisation, initial codes were developed to organise similar fragments of data. NVivo 14 (Lumivero 2023), a qualitative data analysis software, was utilised to conduct this process systematically. Once the entire data set had been coded, researchers (P.V., M.C., V.L.) analysed and clustered codes with similar meanings into potential themes. These themes were analysed independently and in relation to the research question. Codes within the potential themes were reviewed to establish whether they fit coherently into the theme, represent a subtheme, or need to be separated into another theme. Themes were then defined and named to illustrate the central concept of the theme. Following this, writing ensued to illustrate the story within the data and how this answers the research question. This analytic process was recursive, and researchers repeatedly engaged with the data to refine and enhance the validity of themes, resulting in several shifts in themes during the process. It is not unusual for analysis and themes to shift in reflexive thematic analysis, especially whilst writing the final report (Braun and Clarke 2022).

### 3.7 Ethical Considerations

Approval for this study was granted by the WA Country Health Service Human Research Ethics Committee (project reference number RGS0000006301) and the Central Queensland University Human Research Ethics Committee (approval number 0000024004). All participants were given a participant information sheet and provided informed consent. Participants' risk for discomfort and inconvenience was mitigated by scheduling the interview at a date and time of the participants' choosing. Participants were reminded of their freedom to participate and their right to withdraw from the interview at any time without consequence. To maintain confidentiality, participants were asked to select a pseudonym, which was used throughout the interview, although participant numbers are used to report findings.

### 3.8 Rigour and Reflexivity

Rigour was demonstrated using Lincoln and Guba's (1985) criteria of credibility, dependability, confirmability, and transferability. Regular debriefing among team members to challenge ideas throughout the analysis ensured credibility and mitigated researcher bias. Credibility was also established by probing during interviews, a way of member checking, enabling the researcher to repeat and clarify interview responses. Using an audit trail ensured dependability and confirmability, validating that the findings reflected the participants' experiences and not the researchers' (Lincoln and Guba 1985). Transferability was ensured by providing a detailed description of the setting, participants, and the study context. Clearly labelled participant quotations were included to assist the reader in the logic of the analysis and enhance confirmability and credibility. Using an interview guide and conducting the interview with a single researcher ensured consistency and dependability. Reflexivity using memoing was practised throughout the study and was vital in mitigating researcher bias.

## 4 Findings

### 4.1 Participants

Overall, 17 migrant nurses participated in the study. Participants were between 30 and 58 years old, primarily female (*n* = 11) and married (*n* = 14). Participants' source countries were the Philippines (*n* = 7), India (*n* = 6), Kenya (*n* = 1), New Zealand (*n* = 1), Scotland (*n* = 1) and South Africa (*n* = 1). Participant characteristics are detailed in Table 1.

**TABLE 1:** Participant demographics (*n* = 17).

Characteristics	Frequency
Gender	
Male	6
Female	11
Age (years)	
Range	30–58
Mean	37.4
Marital status	
Single	3
Married (without children)	1
Married (with children)	13
Source country	
India	6
Kenya	1
New Zealand	1
Philippines	7
Scotland	1
South Africa	1
Migration status	
First‐time migrant	11
Stepwise migrant	6
Relocation source	
Source country direct to regional area	2
Source country to any other area within Australia (before relocating to the regional area)	9
Host country direct to regional area	4
Host country to any other area within Australia (before relocating to the regional area)	2
Nursing experience (years)	
Range	7–37
Mean	13.4
Length of stay in regional area (years)	
Range	1–7
Mean	3

*Note:* Source countries are where the participants obtained their initial nursing registration. Migration status refers to whether the participants migrated directly from their source country to Australia (first‐time migrant) or another host country (stepwise migrant).

### 4.2 Themes

Seeking meaningful endeavours captures the essence of participants' motivations to migrate to their host country and relocate to a regional area. Participants were usually motivated by multiple factors contributing to their decision to relocate. Participants were seeking meaningful endeavours as nurses, family providers, and individuals. The three subthemes further explain this theme: Pride in nursing, Duty to family, and Personal satisfaction.

#### 4.2.1 Pride in Nursing

Pride was central when participants described their professional motivations for migration. Seeking meaningful endeavours as nurses who show pride in their profession, professional recognition and professional fulfilment were identified as motivations.

##### 4.2.1.1 Professional Recognition

Participants felt ‘proud to be a nurse’ (P10), given that nursing is a ‘four‐year course and we [studied] a lot’ (P9). Then again, participants felt disillusioned after learning that nursing was considered a ‘low‐level’ degree (P6) in their home countries, causing them to be treated as ‘inferior’ to doctors (P9). Despite being university‐educated, the lack of acceptance or treatment as professionals caused participants to question their sense of self‐worth as nurses. P9 stated, ‘There wasn't any use [studying] in a big government college with a scholarship’. This ‘stigma’ prevented participants from utilising their skills, with P9 continuing, ‘We don't get much opportunity to put into practice what we have learned or have a discussion with the doctors’. P7 added, ‘It's a culture that you should wait until the doctors say so’ when discussing the inability to carry out nurse‐initiated interventions. To doctors, participants' education had little to no merit as ‘doctors are superior, nurses are downgraded’ (P16), causing participants to feel ‘scared’ (P16) even when attending regular doctor's rounds.

The ‘stigma’ of nursing being a ‘low‐level’ degree was not limited to how doctors treated participants; instead, it extended to the patients and relatives because ‘[Nursing] is [seen] as a low profession by so many people’ (P16). Participants reported losing sight of their professional roles and passively submitting to patients' demands, ‘because they are the patient, you need to do it for them even if it's not on your job description’ (P14). The service‐oriented nature of nursing often leads others to ‘think [that] a nurse is like a housemaid’ (P16), ‘making [their] work a bit harder than it is supposed to’ (P5).

Participants did not feel a sense of control over their ‘actual’ roles as they often found themselves ‘multitasking’ (P14) and ‘working different positions every day’ (P12), including the role of a ‘dietitian, pharmacist, phlebotomist, ward clerk, or support coordinator’ (P12, P14). Despite undertaking multiple roles, participants reported feeling ‘inferior’ and not being viewed as equals and ‘in the same position [as] other allied health professionals’ (P16).

##### 4.2.1.2 Professional Fulfilment

Participants also felt ‘proud’ of themselves when they saw that their actions contributed to good outcomes for their patients or when they could ‘do things to other people that make them better’ (P15). Participants were motivated when patients showed gratitude, with P10 reporting that gratitude ‘improves my self‐esteem’. Patient feedback and appreciation made participants feel ‘valued’ (P10) and reassured them that they ‘have done a good thing’ (P14). The ‘good feeling that people have appreciated [their] hard work as a nurse’ (P14) reinforced participants' motivations to be ‘always happy to show up and keep working’ (P10).

Participants recognised the importance of their role, with P9 stating, ‘It's good to be a nurse because we can help [the] community’. Participants recognised that being a nurse is ‘a good profession to save and serve the people’ (P16) and reported feeling ‘self‐satisfaction [from] knowing you saved another life’ (P8). On the other hand, when participants were placed in situations where they could not ‘do a good job’ due to patient or working factors, they ‘[felt] useless’ (P7) and started questioning themselves: ‘Why am I doing this? Why am I in nursing’ (P7). This limitation is mainly attributed to workforce and resource shortages such as patient workload where participants ‘were forced to work [in] unsafe [conditions], [such as] looking after a whole ward myself’ (P8) and ‘[having] up to 30 patients’ (P10) because the system is ‘struggling to meet the demands of nursing to patient ratios’ (P2). The inability to ‘focus on one thing at a time’ (P3) prevented participants from functioning to the best of their abilities and ‘giv[ing] quality care’ (P9), making them uneasy that their actions may result in errors or ‘missed care because [we] can't pick what the [patient's] problem is’ (P10). P15 mentioned a ‘typical third world’ setting where the hospital and patients' resources were limited, so they ‘try to be resourceful’ to provide the care their patients require. Participants also reported feeling ‘satisfaction [from] doing great at work’ (P15) and challenge themselves ‘to do [the] job as proper as [I] can’ (P8).

#### 4.2.2 Duty to Family

Seeking meaningful endeavours as family providers, participants' duty to family included filial piety, financial responsibilities, visa security, and family unification and overall safety, which were identified as motivations that contributed to their relocation.

##### 4.2.2.1 Filial Piety

Participants reported motivations to fulfil their duties, obey elders, and meet cultural expectations. P7 stated, ‘[My parents] are expecting something that I should be doing. [It] shouldn't be like that. It's just more [of a] culture’ when describing the need to take up another job to meet ‘family expectations’ (P7). To avoid animosity, P7 stated, ‘I [did it] to lessen their burden’. P6 also admitted to working ‘a lot’ to provide ‘voluntary help for my family’.

Participants' parents or older family members often recommended a nursing degree with the notion that they would pursue their careers abroad. For participants from collectivist cultures, their families had a ‘say’ in their careers and their plans. P14 recalled, ‘[My] parents told me to be a nurse,’ so ‘I just adopted it.’ P14 continued, ‘My father wants Australia; he said it's better to go there than to the Middle East.’ Somewhat, nursing was a way to gain ‘family approval’ like P7, who stated, ‘It's my mum's wish [for me] to become a nurse, so here I am’. Similarly, P5 pursued nursing despite being an ‘aspiring accountant or engineer’ because it ‘was my mum's choice’. Nursing may also be a way to follow their relatives' footsteps, such as P9, who has ‘relatives in [the] nursing background’ or P16, who is ‘proud to be a nurse because all my grandparents [have] big positions [in] the nursing field’.

Nursing being a ‘stable job’ (P6) may be another reason participants were persuaded into the career. ‘Nursing is the best way that you can [go abroad]’ (P12), implying that a nursing degree came with the idea that ‘if you take nursing, then you'll have a secure future’ (P15). Not all participants wanted to leave their home countries, with P3 saying, ‘I feel I'm not being a good daughter [if I leave them behind], but they said you're not going to be able to support us if you're here’. P7 added, ‘It's a very menial pay, [so] there's no way of staying in the Philippines as a nurse.’ The financial obligation was an ‘untold expectation’ (P7) from their parents, where ‘it's just a culture to support parents financially’ (P3) and to ‘be a good help for them in return’ (P6). The financial security that came with a nursing career and parental pressures were enough for participants to push their ‘dreams’ (P7) aside.

##### 4.2.2.2 Financial Responsibilities

Participants described prioritising their family's needs over their own to fulfil their familial responsibilities. Reasonable pay and financial benefits are essential to ‘sustain [a] family’ (P10); hence, as participants assumed the role of a family provider, they sought opportunities to maximise their income after realising their ‘salary won't fit’ (P14). P8 ‘stopped nursing’ after having a daughter to venture into a business whilst looking for options overseas ‘because we don't earn that much’. Participants described not being ‘properly compensated’ (P5) or ‘being paid the bare [amount]’ (P12) for the work that they do. Their salaries were ‘very low’ (P13) and ‘not designed to support a family’ (P15). As participants ‘decided to have a family’ (P14), they were challenged to look for colloquially termed ‘greener pastures’ (P7, P10). In other words, family needs motivated participants to look for opportunities where their ‘skills [are] valued and paid for’ (P10), affording them ‘better lifestyle opportunities’ (P5) and ‘better living’ (P8) so that they can ‘have a meaningful and decent life’ (P10).

##### 4.2.2.3 Visa Security

Aside from financial incentives, participants stated the main reason for relocation to a regional area was to obtain a ‘stronger kind of visa’ (P10) that did not impose many working restrictions and granted them and their families security towards permanent residence. P3 recalled being hired as a ‘part‐time employee’ due to visa restrictions, which only allowed work up to ‘40 h a fortnight’. The inability to work full‐time restricted their incomes, thus limiting their abilities to meet family responsibilities. Participants admitted to relocating to a regional area from other cities because they ‘need sponsorship’ (P14), which was ‘very hard [to get] in the city’ (P3). Although other participants had obtained working visas, some were only temporary visas, which provided uncertainty for when the visa lapsed. Because of this, participants chose to relocate to a regional area ‘because they offered a permanent resident visa’ (P5, P13). P14 described aiming to ‘be a permanent resident to get my daughter here’. Similarly, P11 expressed the same sentiment, ‘aim[ing] towards a permanent resident because we were coming as a family, so whoever offered me that, I was happy’.

##### 4.2.2.4 Family Unification and Overall Safety

Participants had to make certain sacrifices, such as career, psychological, and emotional sacrifices, as they made decisions that prioritised their families' needs over their own. Despite the sacrifices, participants remained motivated by their sense of duty to their families. P10 recalled thinking, ‘Psychologically, am I able to tolerate [moving] as a lone ranger and [leaving] my children’. Participants' emotional well‐being was often put aside as they migrated alone, leaving their families behind temporarily. P14 described feeling ‘alone and sad because [there was] no one to help me’, but that feeling fuelled the motivation to ‘do everything I need to do so that I can get my daughter and husband and we can be together’.

‘Keeping the family together’ (P15) was another motivation for participants, even if it meant sacrificing their careers. P4 remembered having ‘to move around’ to different countries for ‘family reasons’, leading to ‘[not much] growth in my career and [not] being promoted to ‘higher positions'. Similarly, for participants whose husbands had already migrated, it was implied that they would follow them overseas. Participants stated ‘the main reason [for moving] was because my husband was working there’ (P11, P16), had a ‘good job’ (P4), and that ‘[being away] was quite hard on the family, so we all decided to move together’ (P17). More often than not, the countries they first migrated to were not always their first preference for the host country; instead, participants followed what their families had already set in motion. This resulted in some participants migrating again when they ‘had the opportunity’ (P12) or when their initial host country did not fulfil their or their families’ needs, providing them a reason for stepwise or onward migration. These needs may come in the form of political safety, such as being ‘forcibly removed’ (P1) from their home, being ‘scared to walk [in] the road’ (P11), or being unable ‘to go outside because of the [dangerous] behaviour present’ (P13), limiting their quality of life. P12 mentioned wanting the freedom ‘to go anywhere’ and ‘follow the same lifestyle [as] in India’, although this was not always the case in their initial host country. P6 also described living in a ‘very conservative country’ where the movement was restricted, adding it was ‘not the way [I] want to live’.

In addition to political and environmental safety, participants also had to seek educational safety for their children. P13 recalled having to send ‘my children back to India after year 10’ because ‘degree programs are only for [residents]’. The possibility of sending their children back to their source countries for tertiary education risked family separation, so participants sought to migrate to other host countries that provided more educational opportunities for their children. P16 added, ‘I thought [it is] better to move now so the whole family will be [together]'.

#### 4.2.3 Personal Satisfaction

Participants seeking opportunities to improve their financial and working conditions may also fall under this subtheme. However, although these were infrequent, we wanted to discuss other motivations outside of work and family. Personal satisfaction describes participants seeking meaningful endeavours as individuals and refers to motivations to challenge and improve themselves and their lifestyles without the pressures of family and/or work.

##### 4.2.3.1 Improved Lifestyle

Participants reported pressures that motivated them to relocate. P3 reported feelings of not ‘achieving anything’, being ‘left behind’, and feeling ‘pressured’ by friends already overseas as the ‘main motivation’. Participants were also motivated by their personal goals. P5 wanted ‘better career and lifestyle opportunities’, and although there was no pressure to move to a particular host country, social connections remained influential in the choice of the host country. P5 added hearing ‘about this lifestyle, salary, and all those things in Australia’ from a friend and feeling ‘inspired after that’. P6 stated, ‘I need to settle in a better place’ when describing their motivations for relocation.

##### 4.2.3.2 Travel

Only one participant, P2, travelled to seek adventure. Migration was not the initial plan, with P2 stating, ‘[Travelling] was always something I had wanted to do’ and feeling ‘lucky enough to have a gap year and still retain [my] permanent contract [at work]’. Due to P2's source country, there were no ‘additional training or conversion courses' and ‘it was very easy to get my registration and start working’ in the host country during the gap year. P2 was also the only participant who had the opportunity to travel during a ‘gap year,’ so it is difficult to determine whether other participants would have also been motivated to travel if professional and travel regulations did not limit them.

##### 4.2.3.3 Being a Nurse

Aside from seeking opportunities to improve one's lifestyle, some participants found inherent satisfaction in their choice of profession. All participants mentioned a genuine motivation to be involved in caring for others, regardless of whether nursing was their first choice of profession or not. P4 recalled, ‘Initially [my] parents interested me to go for nursing, [but now] working with sick patients makes me happy’. Participants' desire to care for others led them to choose clinical areas where they could make the most difference. For example, P2 chose to work in surgical nursing ‘to see people getting better’ and ‘to see the change in people’. P16 moved from intensive care to maternity because ‘in ICU, I am seeing lots of death, but maternity is a happy place’. Despite changing areas, the desire to care and to nurse remained. Instead of choosing to leave the profession when things became difficult, participants' thoughts were on ‘What area would I want to work in’ not ‘Is there something different I would like to do’ (P2).

Nursing was considered a ‘privilege’ (P17) to help patients improve even in ‘simple ways’ (P14). The ability to be a part of their patients' lives when ‘they're most vulnerable’ (P17) and nursing them ‘back to health’ (P5) offsets the ‘tiring’ (P4), ‘exhausting’ (P8), and ‘distressing’ (P5) nature of the job. However, participants described being demotivated when unable to provide utmost care to their patients. P17 described feeling ‘frustrated because I have this standard, but I could only deliver this standard [because] of the environment’. Participants stated, ‘It's hard to look after the patient when you don't have [resources]’ (P15) and when ‘the working environment is hectic’ (P14). Participants were committed to the profession, so being limited by their working environments motivated them to migrate.

## 5 Discussion

This paper reports research on migrant nurses' intrinsic and extrinsic motivations, some or a combination of which have led them to relocate to their host countries and a regional area. In addition to what is widely published in the literature, which states that nurses are primarily motivated to migrate due to financial, professional, and social factors (Davda, Gallagher, and Radford 2018; Konlan, Lee, and Damiran 2023), the findings in this study provide further insight on motivations which may not always be apparent in other studies. In this section, we discuss extrinsic and intrinsic motivation, including autonomous and controlled motivation, to inform knowledge and translate the findings.

### 5.1 Extrinsic Motivations

#### 5.1.1 Duty to Family

The findings in this study identified financial benefits and visa sponsorship as externally regulated extrinsic motivations, consistent with research reporting finances as a primary motivation for nurse migration (Konlan, Lee, and Damiran 2023). However, financial or externally regulated motivations may not be enough to keep nurses in one host country, as external regulation may often lead to poorer organisational outcomes, such as disengagement and decreased workplace well‐being (Rigby and Ryan 2018). This may explain why some nurses readily leave one host country to proceed to the next, also known as stepwise or onward migration. This occurs when the nurses' arrival in the initial host country is viewed as a mere ‘stepping stone’ whilst they accrue resources and migration experience until they reach their preferred host country (Carlos 2023; Villamin et al. 2024a). This was evident in the participants' experiences of migrating to seek better financial opportunities than in their source countries, followed by their onward migration to another host country when other needs arose, such as professional recognition, family unification, or an improved lifestyle.

Filial piety and meeting family responsibilities were also considered extrinsic motivations. This may stem from the avoidance of guilt (introjected regulation), recognition of the importance of doing one's part in the family (identified regulation), or alignment of doing so with one's goals, values, or identity (integrated regulation). Participants who reported pursuing a nursing degree as this was their family members' wishes were from a collectivist culture, where the needs and goals of their group are prioritised ahead of their own (Triandis 1995). Hence, this study also highlights the importance of considering cultural factors in exploring motivations. Collectivist cultures focus on pursuing group goals over individual interests and value conformity, whilst individualist cultures place individual goals above group goals and value personal freedom and independent decision‐making (Cheng and Liou 2011; Triandis 1995). As such, participants from collectivist cultures reported giving up on their career preferences and pursuing nursing to obey their elders, a controlled motivation due to external pressures from their family and internal pressures to uphold cultural values. This echoes other studies reporting that becoming a nurse is a collective decision (Dahl et al. 2021) and that filial piety plays an important role in the selection of nursing as a career choice among collectivist cultures (Dos Santos 2020). A systematic review of factors influencing youths' career choices identified that parental influence, such as parental pressure and parental professional background, was crucial in career choices among youths in collectivist cultures (Akosah‐Twumasi et al. 2018).

Filial piety is a cultural concept that emphasises adult children's obligations towards their parents to repay a debt of gratitude, upholding their duty to respect and obey their parents (Li, Singh, and Keerthigha 2021; Pan, Chen, and Yang 2022). It may be associated with controlled motivation (introjected regulation); however, it may also be presumed to be autonomous (identified or integrated regulation), a choice or volition, as this study's participants identified and integrated themselves with the importance of upholding their cultural values and expectations. Aligning with filial piety, collectivist cultures also expect to look after their elders financially (Liu 2023; Xiao et al. 2024). This may have contributed to participants selecting a nursing degree as it was often perceived as a stable job and a gateway to migration opportunities, allowing them to provide more financial assistance to their families. Familial financial needs often drove nurse migration stemming from a desire to send remittances and improve their families' economic standing in their source countries (Squires and Amico 2015).

Families are the basic unit of society and are the primary source of emotional support and social capital; thereby, it is not unexpected that families may influence migration decisions (Connell 2014; Hu, Su, and Zhang 2021), which was also identified in this study. Nurses may migrate to certain countries due to perceived educational benefits for their children, perceived lifestyle opportunities where they can permanently settle, or perceived economic benefits to achieve a better quality of life for their families, as done by the participants in this study. Kay and Trevena (2017) reported similar findings when families from Central and Eastern Europe relocated to Scotland, seeking better lives for their families to settle in. Family inclusion is now recognised in employee retention as families influence and shape migration decisions and long‐term settlement (Kay and Trevena 2017; Zhang and Ma 2021).

This study found that visa sponsorship is another extrinsic motivation for regional relocation. Some host countries, such as Australia and Canada, use regional migration schemes that allow employers to sponsor eligible migrants, including nurses, for permanent or temporary migration to designated regional areas or provinces (Government of Canada 2023; Villamin et al. 2023). After arrival in a host country, some migrant nurses may go through complex side‐door or back‐door migration pathways to gain a more stable visa until they gain a pathway to permanent residency (Howe, Stewart, and Owens 2018; Nourpanah 2019). Regional relocation occasionally appeals to migrant nurses as it offers a more straightforward pathway to temporary or permanent residence, as confirmed in this study. However, as with financial benefits, visa sponsorship is considered an external reward; thus, there is still no guarantee that sponsored migrant nurses will remain in the regional area after obtaining residency (Villamin et al. 2023). Nonetheless, migrant nurses may be motivated by the desire to provide stability to their families instead of relocating again, indicating a higher level of motivation in the form of integrated regulation.

#### 5.1.2 Pride in Nursing

These findings demonstrate several professional factors that extrinsically motivate nurses through introjected regulation, such as positive feedback and professional recognition or through identified regulation, such as professional fulfilment. Nurses are motivated when they can perform duties without being restricted by their working environments. Unfavourable work conditions, including a lack of medical or workforce resources or a high workload, may result in missed care (Janatolmakan and Khatony 2022). Missed care refers to incomplete, unmet, or delays in nursing care, which may contribute to moral distress and job dissatisfaction (Janatolmakan and Khatony 2022; Salari et al. 2022). Aside from the working environment, this study found that being limited professionally, in which nurses cannot practice within the scope of their professional training, may lead to feelings of inadequacy and doubt, contributing to nurses seeking career opportunities in other host countries. This finding is consistent with previously published literature on the impact of the working environment, career development, and professional fulfilment as motivations for migration (Konlan, Lee, and Damiran 2023; Toyin‐Thomas et al. 2023). Professional fulfilment can come from the ability to perform one's nursing duties independently, having the freedom to express one's professional opinions, and expanding one's skills and clinical practice (Zhong et al. 2023). A study on stepwise migration and long‐term settlement among Filipino nurses in Singapore showed that career satisfaction and professional recognition were enough for some migrant nurses to choose to stay in Singapore instead of moving to other countries with long‐term settlement policies, such as the UK, USA, or Australia (Carlos 2023). This contradicts the common perception that nurses ‘target’ countries with long‐term settlement policies (Nourpanah 2019; Salami et al. 2014).

#### 5.1.3 Personal Satisfaction

The findings of this study identify personal satisfaction as both an extrinsic and intrinsic motivation. Although the search for better lifestyle opportunities was personally satisfying to the participants, this may still not be classed as intrinsic motivation due to external rewards or internal pressures. Nonetheless, extrinsic motivations of personal relevance are associated with well‐being (Lozano‐Jiménez, Huéscar, and Moreno‐Murcia 2021), highlighting that these may still be effective if they align with personal goals and values.

### 5.2 Intrinsic Motivations

#### 5.2.1 Personal Satisfaction

Some participants were intrinsically motivated to be nurses. Intrinsic motivation represents autonomous motivation and contributes to positive organisational outcomes such as job satisfaction, well‐being, and decreased turnover intention (Toode et al. 2015). However, autonomous motivation is more sensitive to work‐related aspects and, thus, may still diminish and be replaced by more controlled forms of motivation as negative work‐related factors such as exhaustion, overload, and burnout accumulate (Fernet et al. 2021; Toode et al. 2015). Although intrinsically motivated nurses may still find satisfaction and meaning despite unsatisfactory working environments, threats to psychological energy may result in a decreased quality of motivation (Austin et al. 2020). This may explain why despite being intrinsically motivated, participants still left their source countries and sought better working environments that fuelled their intrinsic and extrinsic motivations. The other intrinsic motivation reported in this study was relative to the participants' satisfaction, such as travel. This was considered an intrinsic motivation, as this was undertaken for enjoyment and interest in the activity.

Integrated regulation has been excluded from motivation studies in the context of work due to very similar correlations to identified and intrinsic regulations (Lohmann et al. 2017; Van den Broeck et al. 2021). Thus, this study did not report findings on integrated regulation in the work context.

### 5.3 Autonomous and Controlled Motivation

Different levels of motivation may result in varied long‐term outcomes, as nurses who were motivated to migrate by lower levels of extrinsic motivation can experience and deal with migration challenges differently from nurses who have migrated for reasons that match their goals or as a matter of personal importance. The findings in this study may provide another perspective as to why some migrant nurses stay whilst others continue to migrate.

Scholars have combined external and introjected regulation to form a controlled motivation composite and combined identified, integrated, and intrinsic regulation to form an autonomous motivation composite (Gagné and Deci 2005; Ryan and Deci 2000). Autonomous motivation is characterised by individuals engaging in an activity, fully endorsing their actions, whilst controlled motivation is characterised by individuals engaging in an activity due to external rewards, pressures, or power dynamics (Deci, Olafsen, and Ryan 2017). Autonomous motivation predicted role perseverance among nurse clinical instructors (Asher‐Slimak, Warshawski, and Barnoy 2023) and occupational commitment and turnover intention among nurses (Fernet et al. 2021). Controlled motivation predicted occupational and organisational turnover among newly graduated nurses (Fernet et al. 2017).

Multiple motivations influence intentional behaviours simultaneously, and extrinsic and intrinsic motivations may synergistically affect nurses' intentions to stay (Ryan and Deci 2020; Satoh, Watanabe, and Asakura 2018). Therefore, it may be prudent to focus on supporting nurses in achieving and maintaining more autonomous forms of motivation through work environments that enable them to collectively meet their personal, professional, and familial goals rather than solely focusing on external rewards or incentives. Work environments where nurses can practice within their scope and perform their duties in a way that is fulfilling to them whilst meeting their family responsibilities through reasonable pay or benefits may support nurses in remaining highly motivated.

**FIGURE 1:**
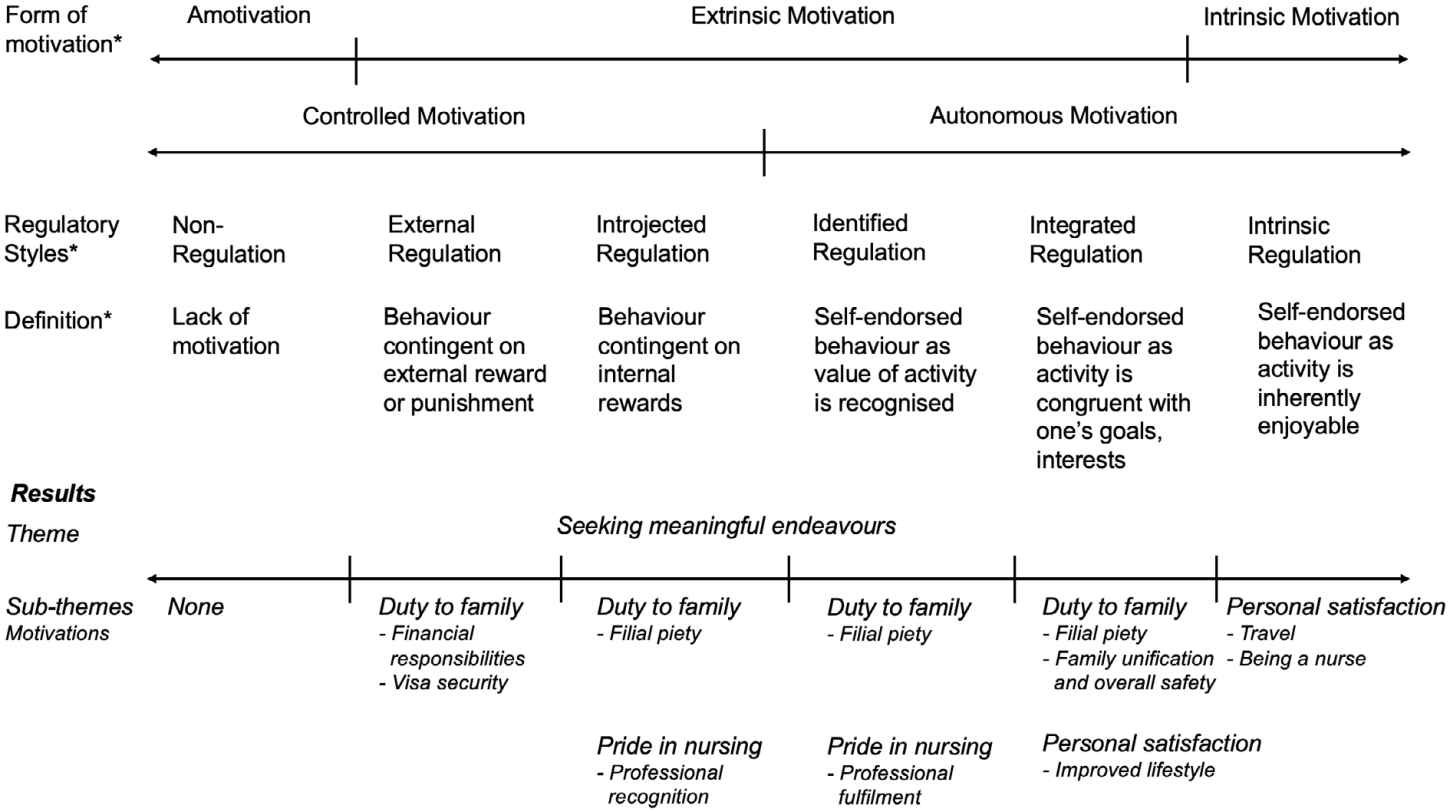
Migrant nurses' intrinsic and extrinsic motivations. Figure 1 summarises participants' intrinsic and extrinsic motivations along the motivation continuum. The * represents text adapted from Cunningham et al. (2022); Gagné and Deci (2005); and Ryan and Deci (2000).

Figure 1 displays the motivation continuum of SDT described by Ryan and Deci (2000), along with a summary of this study's findings.

### 5.4 Strengths and Limitations

Study limitations include the sample size and one hospital setting in a regional geographic location; hence, it cannot be assumed that findings will be similar in other places. The study population included migrant nurses; however, the findings may not be generalisable to other migrant professionals, migrant skilled workers, or non‐migrant nurses.

Despite this, the study has several strengths. The research design allowed the researchers to stay close to participants' perceptions and experiences, providing extensive knowledge that may influence interventions, inform policy, and instigate change (Bradshaw, Atkinson, and Doody 2017; Chafe 2017; Villamin et al. 2024b). Semi‐structured interviews also increased the depth of the data being gathered, including exploring and clarifying participant responses, with participants discussing all interview topics. Using strategies to achieve rigour supported reliable and trustworthy findings (Lincoln and Guba 1985) providing nuanced insights and contributing to new knowledge.

### 5.5 Recommendations for Future Research

With the increase in nurse migration from both collectivist and individualist cultures, there is an opportunity to study the motivations of migrants from individualist cultures. Additionally, apart from being all migrants, the participants in this study were quite heterogeneous in their source countries and migration experience. Toode et al. (2015) identified a positive correlation between age and duration of service with introjected regulation, highlighting that more tenured nurses tend to be motivated extrinsically rather than intrinsically over time. This may imply that a shift in participant demographics may yield different findings. There is an opportunity to conduct research with more homogenous samples, including stepwise migrants from various sources and/or host countries. Future research could also examine the impact and engagement of migrant nurses from the perspectives of the host country.

The findings of this study emphasised filial piety in influencing migrant nurses' decision to become nurses. Further research could explore the long‐term impacts of filial piety, such as occupational turnover among nurses who initially had different career aspirations, its effects on overall job satisfaction, or its influences on one's nursing career trajectory and professional growth.

Additionally, this study demonstrates the merit of using SDT for studies on motivation. This may serve as a foundation for larger‐scale studies employing the SDT perspective on studies on the broader workforce retention, motivation, or work engagement in healthcare. Further quantitative and longitudinal studies on migrant nurses' motivations may support and expand on this study's findings.

### 5.6 Implications for Policy and Practice

This research reports on migrant nurses' intrinsic and extrinsic motivations along the motivation continuum, expanding knowledge on experiences that contribute to migration. For the source countries, understanding that nurses are motivated by a combination of intrinsic and extrinsic factors may aid them in creating working environments that support autonomous motivation. These may include designing professional environments wherein nurses are well‐regarded in their roles and can practice according to their professional training and qualifications. These may also include reviewing benefits and providing reasonable pay that enables nurses to support their families' needs and provide a good lifestyle for their families. Working conditions such as these may foster more autonomous forms of motivation, which, in turn, may allow nurses to weigh in on their overall conditions and look for more reasons to stay rather than leave their source countries.

Similarly, organisations and policymakers of host countries may develop tailored policies and strategies to create conditions that support autonomous motivations among migrant nurses. This may contribute to workforce retention among migrant nurses (Villamin et al. 2023), increasing the workforce capacity available to the community. Migrant nurses who are highly motivated may be more resilient to migration and transition challenges and, therefore, may readily adapt and transition into the workforce.

## 6 Conclusion

Migrant nurses' motivations to migrate are complex and go beyond financial and economic benefits. They may be motivated by financial responsibilities, filial piety, and family unification and overall safety, all of which align with their duty to family as a primary motivation. Professional motivations to migrate include seeking professional development, recognition, and improved working conditions. Migrant nurses may simultaneously be motivated by intrinsic and extrinsic factors, so positive migration outcomes may be achieved by creating environments that support autonomous rather than controlled motivation. This may be achieved by organisations focusing on developing environments supporting professional recognition and fulfilment rather than increasing financial rewards. Although financial incentives are beneficial, the motivations these provide are controlled and may not lead to long‐term engagement and workplace retention. Work environments that enable migrant nurses to practice within the scope of their professional training and perform their duties in a fulfilling manner whilst meeting their family obligations through reasonable pay or benefits may support nurses in remaining highly or autonomously motivated, which may contribute to retention, work engagement, and job satisfaction.

## Author Contributions

P.V., M.C., V.L. made substantial contributions to conception and design, or acquisition of data or analysis and interpretation of data. P.V., M.C., V.L., D.K.T. involved in drafting the manuscript or revising it critically for important intellectual content. P.V., M.C., V.L., D.K.T. gave final approval of the version to be published. Each author should have participated sufficiently in the work to take public responsibility for appropriate portions of the content. P.V., M.C., V.L., D.K.T. agreed to be accountable for all aspects of the work in ensuring that questions related to the accuracy or integrity of any part of the work are appropriately investigated and resolved.

## Conflicts of Interest

The authors declare no conflicts of interest.

## Data Availability

The authors have nothing to report.
